# Supplementary material for: Searching for the Achilles’ Heel of Urethane Linkage—An Energetic Perspective
Source: Polymers (Basel). 2024 Apr 17;16(8):1126. doi: 10.3390/polym16081126 (PMC11053941; doi:10.3390/polym16081126)
Supplement: Supplementary file 1 [file polymers-16-01126-s001.zip › polymers-2885470-supplementary.pdf]

polymers

MDPI

Searching for the Achilles' Heel of Urethane Linkage—An Energetic Perspective

Tamás Horváth [1,*], Karina Kecskés [1], Anikó Jordán Csábrádiné [1], Emma Szőri-Dorogházi [1], Béla Viskolcz [2] and Milán Szőri [1,*]

[1] Institute of Chemistry, University of Miskolc, Miskolc-Egyetemváros A/2, H-3515 Miskolc, Hungary; kecskes.karina@student.uni-miskolc.hu (K.K.); aniko.jordan@uni-miskolc.hu (A.J.C.); emma.szori-doroghazi@uni-miskolc.hu (E.S.-D.)
[2] Higher Education and Industrial Cooperation Centre, University of Miskolc, H-3515 Miskolc, Hungary; bela.viskolcz@uni-miskolc.hu
* Correspondence: tamas.horvath@uni-miskolc.hu (T.H.); milan.szori@uni-miskolc.hu (M.S.)

Supplementary Information

Table S1. Reaction enthalpy values (in kJ/mol) for the termination of urethane linkage by different hydrogenation pathways in gas phase as well as in aniline and aqueous phases.

reactant	$\Delta_{R;298.15K}H^0$ (kJ/mol)			$\Delta_{R;398.15K}H$ (kJ/mol)		
	gasphase (ε=1)	aniline (ε=6.8)	water (ε=78.4)	gasphase (ε=1)	aniline (ε=6.8)	water (ε=78.4)
H_2 (TS**a**)	-0.4	-0.7	-5.3	-2.2	-2.5	-7.0
H_2 (TS**b**)	-51.5	-52.6	-60.1	-2.2	-54.4	-61.8
H_2 (TS**c**)	20.0	17.0	13.3	17.3	14.3	10.7
H_2 (TS**d**)	-22.8	-35.0	-42.7	-1.4	-13.7	-21.5

Table S2. Activation enthalpy values (in kJ/mol) for the termination of urethane linkage by different hydrogenation pathways in gas phase as well as in aniline and aqueous phases.

reactant	$\Delta_{TS;298.15K}H^0$ (kJ/mol)			$\Delta_{TS;398.15K}H$ (kJ/mol)		
	gasphase (ε=1)	aniline (ε=6.8)	water (ε=78.4)	gasphase (ε=1)	aniline (ε=6.8)	water (ε=78.4)
H_2 (TS**a**)	317.4	291.2	274.6	314.8	288.6	272.0
H_2 (TS**b**)	405.6	359.3	346.2	403.3	356.9	343.7
H_2 (TS**c**)	431.4	357.8	314.7	429.5	356.0	312.9
H_2 (TS**d**)	296.2	289.9	281.5	317.0	310.9	302.4

Table S3. Zero-point corrected energy of the reaction ($\Delta_R E^0$ in kJ/mol) and zero-point corrected energy of the activation ($\Delta_{TS}E^0$ in kJ/mol) for the termination of urethane linkage by different hydrogenation pathways in gas phase as well as in aniline and aqueous phases.

reactant	$\Delta_R E^0$ (kJ/mol)			$\Delta_{TS}E^0$ (kJ/mol)		
	gasphase (ε=1)	aniline (ε=6.8)	water (ε=78.4)	gasphase (ε=1)	aniline (ε=6.8)	water (ε=78.4)
H_2 (TS**a**)	2.9	2.3	-2.7	325.0	298.8	281.6
H_2 (TS**b**)	-48.7	-50.0	-57.7	412.4	366.4	353.1
H_2 (TS**c**)	26.7	23.7	19.6	437.5	363.0	319.9
H_2 (TS**d**)	2.9	-9.4	-16.5	326.0	319.4	310.9

Table S4. Reaction enthalpy values (in kJ/mol) for the termination of urethane linkage by different reactants in gas phase as well as in aniline and aqueous phases.

reactant	$\Delta_{R;298.15K}H^0$ (kJ/mol)			$\Delta_{R;398.15K}H$ (kJ/mol)		
	gasphase (ε=1)	aniline (ε=6.8)	water (ε=78.4)	gasphase (ε=1)	aniline (ε=6.8)	water (ε=78.4)
H_2 (TS**a**)	-0.4	-0.7	-5.3	-2.2	-2.5	-7.0
H_2O (TS**e**)	2.1	6.8	7.7	1.0	5.6	6.5
CH_3OH (TS**f**)	9.1	13.1	14.2	9.2	13.3	14.3
H_2O_2 (TS**g**)	-4.1	7.2	14.7	-3.8	-6.7	14.1
$C_2H_6O_2$ (TS**h**)	22.0	23.5	22.8	22.1	23.6	22.9
NH_3 (TS**i**)	1.8	1.7	-1.9	1.4	1.5	-2.1
CH_3NH_2 (TS**j**)	-4.4	-9.4	-15.5	-3.9	-8.9	-14.9
$C_2H_7O_3P$ (TS**k**)	14.1	17.0	18.2	14.3	17.2	18.3

Table S5. Activation enthalpy values (in kJ/mol) for the termination of urethane linkage by different reactants in gas phase as well as in aniline and aqueous phases.

reactant	$\Delta_{TS;298.15K}H^0$ (kJ/mol)			$\Delta_{TS;398.15K}H$ (kJ/mol)		
	gasphase (ε=1)	aniline (ε=6.8)	water (ε=78.4)	gasphase (ε=1)	aniline (ε=6.8)	water (ε=78.4)
H_2 (TS**a**)	317.4	291.2	274.6	314.8	288.6	272.0
H_2O (TS**e**)	224.9	207.8	194.0	223.7	206.5	192.7
CH_3OH (TS**f**)	206.8	190.6	179.4	206.0	189.7	178.4
H_2O_2 (TS**g**)	174.8	160.5	150.2	174.3	152.8	149.0
$C_2H_6O_2$ (TS**h**)	202.9	189.4	180.1	201.6	188.0	178.7
NH_3 (TS**i**)	237.3	212.9	202.4	236.3	211.7	201.2
CH_3NH_2 (TS**j**)	218.6	192.0	182.1	218.1	191.3	181.4
$C_2H_7O_3P$ (TS**k**)	302.4	287.2	267.1	302.2	287.1	267.1

Table S6. Zero-point corrected energy of the reaction ($\Delta_R E^0$ in kJ/mol) and zero-point corrected energy of the activation ($\Delta_{TS} E^0$ in kJ/mol) for the termination of urethane linkage by different reactants in gas phase as well as in aniline and aqueous phases.

reactant	$\Delta_R E^0$ (kJ/mol)			$\Delta_{TS} E^0$ (kJ/mol)		
	gasphase (ε=1)	aniline (ε=6.8)	water (ε=78.4)	gasphase (ε=1)	aniline (ε=6.8)	water (ε=78.4)
H_2 (TS**a**)	2.9	2.3	-2.7	325.0	298.8	281.6
H_2O (TS**e**)	4.3	9.4	10.2	229.3	212.7	198.5
CH_3OH (TS**f**)	9.0	13.4	14.3	210.4	194.6	183.1
H_2O_2 (TS**g**)	-5.1	8.6	16.6	177.3	164.0	154.4
$C_2H_6O_2$ (TS**h**)	22.4	24.2	22.7	208.1	194.2	184.6
NH_3 (TS**i**)	3.7	3.2	0.1	242.1	217.7	207.5
CH_3NH_2 (TS**j**)	-5.2	-10.2	-16.7	221.9	195.7	185.7
$C_2H_7O_3P$ (TS**k**)	14.5	16.8	18.8	305.5	288.3	269.1

Table S7. Reaction enthalpy values (in kJ/mol) for the termination of urethane linkage by different ionization methods in gas phase as well as in aniline and aqueous phases.

reactant	$\Delta_{R:298.15K} H^0$ (kJ/mol)			$\Delta_{R:398.15K} H$ (kJ/mol)		
	gasphase (ε=1)	aniline (ε=6.8)	water (ε=78.4)	gasphase (ε=1)	aniline (ε=6.8)	water (ε=78.4)
$MPCate^-$	101.0	-68.8	-116.3	102.0	-68.1	-72.5
$MPCate^+$	791.0	617.4	594.5	791.2	617.4	594.5
H^+ (1)	-833.4	-541.0	-496.1	-826.7	-1027.0	-497.9
H^+ (2)	-951.0	-476.2	-391.0	-843.0	-1030.5	-505.2
OH^- (**1**)	97.2	75.5	55.6	96.6	74.8	55.1
OH^- (**2**)	13.4	13.1	6.9	13.4	13.1	7.1

Table S8. Zero-point corrected energy of the reaction ($\Delta_R E^0$ in kJ/mol) for the termination of urethane linkage by electron capture/detachment in gas phase as well as in aniline and aqueous phases.

reactant	$\Delta_R E^0$ (kJ/mol)		
	gasphase (ε=1)	aniline (ε=6.8)	water (ε=78.4)
$MPCate^-$	99.2	-69.7	-113.6
$MPCate^+$	790.8	617.5	594.6
H^+ (1)	-828.3	-535.5	-490.5
H^+ (2)	-944.4	-469.5	-384.2

Table S9. Activation enthalpy values (in kJ/mol) for the termination of urethane linkage by different hydroxylation methods in gas phase as well as in aniline and aqueous phases.

reactant	$\Delta_{TS;298.15K}H^0$ (kJ/mol)			$\Delta_{TS;398.15K}H^0$ (kJ/mol)		
	gasphase (ε=1)	aniline (ε=6.8)	water (ε=78.4)	gasphase (ε=1)	aniline (ε=6.8)	water (ε=78.4)
OH^- (**1**)	130.3	128.4	129.9	129.1	127.2	128.9
OH^- (**2**)	164.4	134.1	105.2	163.5	133.2	104.5

Table S10. Zero-point corrected energy of the reaction ($\Delta_R E^0$ in kJ/mol) and zero-point corrected energy of the activation ($\Delta_{TS} E^0$ in kJ/mol) for the termination of urethane linkage by different hydroxylation methods in gas phase as well as in aniline and aqueous phases.

reactant	$\Delta_R E^0$ (kJ/mol)			$\Delta_{TS} E^0$ (kJ/mol)		
	gasphase (ε=1)	aniline (ε=6.8)	water (ε=78.4)	gasphase (ε=1)	aniline (ε=6.8)	water (ε=78.4)
OH^- (**1**)	100.8	79.3	59.1	134.4	133.2	133.7
OH^- (**2**)	14.2	14	7	168.2	138.1	108.1
